# Innovative health care mobility services in the US

**DOI:** 10.1186/s12889-020-08803-5

**Published:** 2020-06-11

**Authors:** Mary K. Wolfe, Noreen C. McDonald

**Affiliations:** Department of City & Regional Planning, The University of North Carolina at Chapel Hill, New East Building, CB 3140, Chapel Hill, NC 27599-3140 USA

**Keywords:** Access to health care, Health care transportation, Shared mobility, Non-emergency medical transportation, NEMT, Ridesourcing, Ridehailing, TNCs

## Abstract

**Background:**

Transportation barriers prevent millions of people from accessing health care each year. Health policy innovations such as shared savings payment models (commonly used in accountable care organizations) present financial incentives for providers to offer patient transportation to medical care. Meanwhile, ridesourcing companies like Uber and Lyft have entered the market to capture a significant share of spending on non-emergency health care transportation. Our research examines the current landscape of innovative health care mobility services in the US.

**Methods:**

We conducted an environmental scan to identify case examples of utilization of ridesourcing technology to facilitate non-emergency health care transportation and developed a typology of innovative health care mobility services. The scan used a keyword-based search of news publications with inductive analysis. For each instance identified, we abstracted key information including: stakeholders, launch date, transportation provider, location/service area, payment/booking method, target population, level of service, and any documented outcomes.

**Results:**

We discovered 53 cases of innovation and among them we identified three core types of innovation or collaboration. The first and most common type of innovation is when a health care provider leverages ridesourcing technology to book patient trips. This involves both established and nascent transportation companies tailoring the ridesourcing experience to the health care industry by adding HIPAA-compliance to the booking process. The second type of innovation involves an insurer or health plan formally partnering with a ridesourcing company to expand transportation offerings to beneficiaries or offer these services for the first time. The third type of innovation is when a paratransit provider partners with a ridesourcing company; these cases cite increased flexibility and reliability of ridesourcing services compared to traditional paratransit.

**Conclusions:**

Ridesourcing options are becoming a part of the mode choice set for patients through formal partnerships between ridesourcing companies, health care providers, insurers, and transit agencies. The on-demand nature of rides, booking flexibility, and integration of ride requests and payment options via electronic medical records appear to be the strongest drivers of this innovation.

## Background

### Access to health care

Health care transportation refers to any transportation to medical facilities that is non-emergency in nature (e.g. to medical appointments, to an urgent care facility, or being discharged from the hospital). When patients have access to routine and preventative care, overall health outcomes are improved and costly ambulance bills or emergency department visits can be avoided. Delays in medical treatment can lead to progression of chronic disease and ultimately, poorer health outcomes and excessive use of resources [1]. In a recent study, we estimated that 5.8 million people in the US delayed non-emergency medical care due to lack of transportation in 2017 [2].

A systematic review concluded that transportation barriers are a significant impediment to health care access, especially for people with lower incomes or those who are underinsured or uninsured. Such barriers often include lack of access to a vehicle [3]. Neighborhoods with access to public transportation commonly rely on aging transportation infrastructure, unreliable service, or fixed routes that do not align with the location of health care facilities. In many cases, riding the bus or the subway can be physically challenging for people with disabilities, chronic illness, or obesity.

The US has seen a proliferation and normalization of shared mobility technology in recent years; it is estimated that 36% of Americans used some form of ridesourcing service in 2018 [4]. There is federal recognition of the swiftly evolving landscape of shared mobility as transit agencies grapple with opportunities presented by these technological advancements. The US Department of Transportation has sponsored research and pilot projects aimed at exploring partnerships between transit and shared mobility providers [5, 6]. Ridesourcing companies like Uber and Lyft have entered the market to capture a significant share of current spending on non-emergency health care transportation [7] and health care providers are leveraging shared mobility services to establish new ways for patients to access on-demand rides to and from medical appointments.

This paper examines the current landscape of these innovative health care mobility services. We first describe the policy environment in which innovation is occurring. We then illustrate and catalog mobility services by key features and provide specific case examples of hospitals, health systems, and paratransit providers who are leveraging ridesourcing technology to improve service delivery of health care transportation.

### Traditional provision of health care transportation

For many people, driving oneself, getting a ride from a friend or family member, taking public transportation, or ordering a taxi are viable modal options to travel to health care facilities and medical appointments. For individuals with mobility- or financial-related barriers, such as lack of a personal vehicle, there are various specialized transportation options for such trips. We describe several of these alternatives here.

*Paratransit,* in the broadest sense, refers to flexibly scheduled and flexibly routed passenger transportation that supplements fixed-route systems run by public transit agencies. The 1990 Americans with Disabilities Act (ADA) requires that transit operators provide accessible paratransit service (often called ‘ADA complementary’). While paratransit services are commonly perceived as a dedicated service for elderly riders and riders with disabilities, a range of paratransit services exists serving all rider types. Paratransit serves a number of trip purposes, with health care-related trips among them. In many cases, the service is funded by 5310 and 5311 formula grants, which are transportation funding opportunities passed from federal to state to local governments with the aim of reducing operational and capitals costs of transit providers. Paratransit can be provided by both public transportation agencies and other (private or not-for-profit) entities. Paratransit modes can include demand-responsive buses, van services, hospital and care provider-based shuttles, and vehicles for hire including livery vehicles and taxis.

Beyond paying for one’s own health care transportation, there are various programs to help pay for the cost of these trips. *Medicaid non-emergency medical transportation (NEMT)* is a Medicaid benefit that facilitates access to and from medical services for beneficiaries who have no means of transportation*,* or who need accommodations for physical or intellectual disabilities. Since its inception in 1966, Medicaid pays for NEMT services using the most appropriate and least costly form of transportation. Through this required benefit, states purchase hundreds of millions of rides from taxis, livery vehicles, vans, ambulettes, and public transit every year. Although comprehensive data about Medicaid NEMT expenditures do not exist because states are not required to separately report on this item, the Transit Cooperative Research Program estimates NEMT spending at $3 billion annually, which is less than 1% of total Medicaid expenditures [8].

The majority of states have evolved to deliver NEMT through NEMT brokers or managed care organizations (MCOs). In most of these states, the broker or MCO receives a per capita payment to manage the NEMT benefit.^1^ A few states directly fund government entities such as departments of transportation to provide NEMT while others deliver NEMT on a fee-for-service basis through local service providers. Some jurisdictions provide gas cards or bus passes to beneficiaries. Administration of NEMT services is a significant logistical undertaking for state Medicaid programs.

Beyond Medicaid, health care transportation is becoming more prevalent in other federal programs and health insurance markets. Traditional Medicare covers NEMT via ambulance only^2^; however, NEMT has become a popular supplemental benefit in the *Medicare Advantage (MA)* program. In 2016, NEMT was available to roughly 25% of MA’s 19 million enrollees [9]. In May 2018, the Centers for Medicare & Medicaid Services issued a final rule on a new policy as part of a broad 2019 Medicare payment rule that gives MA plans greater flexibility in choosing supplemental benefits offered to enrollees with chronic illness; nonmedical benefits can include ridesourcing services. In 2019, about 22 million Americans were enrolled in MA plans, which was slightly greater than one third of all Medicare beneficiaries [10].

The *US Department of Veterans Affairs (VA)* offers mileage reimbursement and transportation services for travel to medical and rehabilitation appointments for veterans with disabilities who meet at least one of their qualifying criteria. The VA’s Veterans Transportation Program offers travel solutions to and from VA health care facilities at little or no cost to eligible veterans.

In 2010, the Patient Protection and Affordable Care Act brought about comprehensive health care reform including provisions and programs to test and expand new models of delivering and paying for care, such as the creation of *accountable care organizations (ACOs).* Under the ACO concept, health care providers are organized into teams that together are responsible for the health of a given patient population and the cost of providing its care. ACOs receive bonuses for meeting quality and cost targets while in some cases incurring penalties for falling short of targets. Some ACOs provide beneficiaries with transportation, recognizing that it is one of many strategies to address social needs that have an impact on health, commonly referred to as social determinants of health.

*Charitable support* is also an important supplier of health care transportation in many communities. Community volunteer services, such as those organized through faith-based groups, often provide assistance through a supply of volunteer drivers. Large not-for-profit organizations also offer funding for transportation to care, such as CancerCare, an organization that provides financial assistance for treatment-related transportation to people affected by cancer. In some cases, hospitals and other care facilities have ad-hoc, charity-based funds to facilitate transportation.

### Dynamic policy context

Important policy shifts have occurred which directly (and indirectly) shape the context in which people seek out transportation to health care. Several health care delivery system reforms of the Affordable Care Act were mentioned above; here we will briefly discuss amendments to the Anti-Kickback statute as well as Medicaid waivers.

Effective January 2017, the Department of Health and Human Services and the Office of Inspector General issued a federal Safe Harbor ruling, changing the system of the provision of medical transportation [11]. Prior to this, the Anti-Kickback Statute, originally enacted by the Office of Inspector General in 1972, stated that no health care provider or institution receiving federal dollars could offer anything of financial value that may increase referrals for their publicly- or privately-insured patients; these “inducements” yielded criminal penalties and substantial fines. This criminal statute was intended to protect patients and federal health care programs from fraud and abuse. The 2017 ruling amended this statute by adding new safe harbors that protect certain payment practices and business arrangements from sanctions, making it permissible for eligible medical providers—including hospitals, clinics, physician’s offices, dialysis clinics, medical laboratories, and physical therapists—to offer or facilitate transportation for established patients.

By providing protection for health care entities from penalties related to a possible conflict of interest should they want to include medical transportation as part of their benefit package, the Safe Harbor ruling opened the door for various entities to get involved in medical transportation without fear of legal repercussions. With this change, overall volume of medical trips may increase due to the fact that health care providers can now offer transportation to members who are not covered by Medicaid and who previously did not receive a transportation benefit. Health care providers can contract with taxi companies, mobility companies, or provide transportation in-house.

Another relevant policy shift relates to how the federal government and some states have been reexamining the Medicaid NEMT benefit [12]. As mentioned above, NEMT is a mandatory benefit for Medicaid beneficiaries; however, since 2017 states can limit its availability through federal waivers as Medicaid enters a period of experimentation and potentially reduced federal resources [13]. These Section 1115 Medicaid demonstration waivers allow states to test new approaches in Medicaid that differ from federal program rules. While state Medicaid Agencies navigate decisions about Medicaid waivers, there is evidence to support that NEMT is a worthwhile investment. The state of Florida commissioned an independent evaluation of its NEMT program and found that every dollar invested in the services saved $11.08 in avoidable hospitalization costs, which is equal to a return on investment of more than 1100% [14].

## Methods

This research documents the current landscape of innovation in health care transportation services. The aim of our study was to identify examples of ways in which new ridesourcing services are changing existing modes of access to medical care and providing new ways for patients to reach health care facilities. We used publicly available and subscription search engines to locate news-based sources describing instances in which ridesourcing technology is being leveraged by an institution to facilitate health care transportation in the US.

We identified innovative examples of ridesourcing use in health care transportation by conducting an environmental scan. Environmental scans originated in business but are increasingly used in public health research; they are used to gather knowledge and identify shifts related to social, economic, and technological contexts [15]. Researchers have conducted environmental scans to identify innovations in decision-making training programs for health professionals [16, 17]; to evaluate the landscape of health care access quality measurement [18]; and to examine electronic consultation services between primary care providers and specialists available worldwide [19]. Environmental scans leverage some of the key features of systematic review protocol, including clearly-defined search parameters and predetermined inclusion criteria, and are well-suited for emerging topics where the academic literature is not well-developed enough to support a systematic review.

### Terminology

Shared mobility refers to any mode, whether bicycle, car, public transit, or other mode, in which shared use by multiple users (concurrent or sequential) is often facilitated by smartphone apps and technology [20]. Ridesourcing, or ridehailing, has become one of the most recognized forms of shared mobility. Ridesourcing companies, or Transportation Network Companies (TNCs), such as Uber and Lyft, are defined for regulatory purposes as companies that use an online-enabled platform to connect passengers with drivers who use their personal, non-commercial vehicle to provide trips [21]. We use the term “innovative” in this scan to describe a departure from traditional provision of health care transportation (as described earlier).

### Search strategy

We employed a keyword-based search of news articles, news transcripts, web-based publications, and press releases published from January 1, 2005 to January 31, 2018 to identify innovative case examples in the US in which ridesourcing technology was utilized to connect patients to trips in a vehicle for non-emergency medical purposes. Our search parameters were: (ridehailing OR ridesourcing OR TNC OR Uber OR Lyft) AND (health OR medical OR NEMT) AND (transportation AND health AND medical). These terms were used to search Google News and LexisNexis. We utilized a search feature that excluded duplicates in the display of results.

### Case selection & evidence synthesis

For each article identified, we reviewed the title to determine relevance. We then reviewed each relevant article in full according to our inclusion criteria: case examples in the US in which ridesourcing technology was utilized to connect patients to trips in a vehicle for non-emergency medical purposes. From articles that reported on cases meeting these inclusion criteria, we selected specific cases with the most complete reporting of information and abstracted the following characteristics: key stakeholders involved, launch date, transportation provider, location and service area, who pays for service, booking method, payment method, target population, level of service, and any documented outcomes of the service thus far.

We qualitatively analyzed this information to create a typology of innovative health care mobility services. Specifically, we examined the key stakeholders and booking method of each case to discern important differences and similarities among cases. Understanding who is involved in the ride arrangement and who, specifically, books the ride in each case was the most effective way to analyze the case examples. This process was iterative and followed an inductive approach; we identified patterns, resemblances, and regularities across cases to generate our final typology.

## Results

Our search yielded 3321 publications. We excluded industry trade press publications (*n* = 2293) and blogs (*n* = 491) leaving 537 publications. After reviewing newswires and press releases (*n* = 224), news transcripts (*n* = 207), newspapers (*n* = 74), and other web-based publications (*n* = 32), we discovered 53 cases that met our inclusion criteria (i.e. case examples in the US in which ridesourcing technology was utilized to connect patients to trips in a vehicle for non-emergency medical purposes). After analysis, we identified three core types of innovation: 1) When a health care provider leverages ridesourcing technology; 2) When an insurer partners with a TNC; and 3) When a paratransit provider partners with a TNC. This section describes the structure of each type of innovation and includes an example of each and the typology is summarized in Table 1.

**Table 1:** Typology of Innovative Health Care Mobility Services

	*Type I*	*Type II*	*Type III*
	Health care provider leverages TNC technology	Insurer partners with TNC	Paratransit provider partners with TNC
**Who books the ride?**	Clinician (on patient’s behalf); patient	Patient or clinician	Usually the rider/patient
**Who pays?**	Health care provider; broker; patient	Insurance company; health plan	Transit agency; patient pays ‘fare’ with substantial subsidy from transit agency
**Eligible for Medicaid reimbursement?**	Varies; in many cases, yes, given patient eligibility	n/a	Yes, given patient eligibility
**Patient Benefits:**	• Shorter wait times & less uncertainty • Reminders through smartphone or analog phone	• Financial support • Addresses social determinant of health	• Flexible booking circumvents need for advance booking • Increased trip reliability • Patients who otherwise can’t afford TNC service have access
**Health Care Provider Benefits:**	• Real-time tracking patients’ trips as well as own spending • Flexible booking	• Greater patient engagement • Reduced costs in long-term	• Reduced appointment no-shows

Source: authors’ own analysis of findings of nationwide scan

### Type I: health care provider leverages ridesourcing technology

The first type of innovation is when a health care provider leverages ridesourcing technology to book patient trips. This was the most common type of innovation we found and it primarily involves transportation companies tailoring the ridesourcing experience to the health care industry. The critical feature of this innovation is the added HIPAA compliance of the booking process. Health care associates can order rides for patients from new and existing ridesourcing services through a HIPAA-compliant web platform. Access to this platform occurs through digital integration of a web tool built into a provider’s existing system or as a third-party platform. This allows for the transportation booking process to be digitally integrated with electronic medical records (EMRs) while safeguarding protected health information and maintaining HIPAA compliance. These centralized transportation booking platforms, or dashboards, allow providers to track patients’ trips, record billing and spending information, and send patient reminders to a mobile or landline. Importantly, providers can schedule rides on behalf of patients, which is most essential for patients without a smartphone. As the most common type of innovation we identified, the level of formality of these arrangements varied. In some cases, hospitals simply posted a Lyft discount code in the discharge area while in other cases, a full-scale business line was launched, as was the case with Uber Health.

### Example of type I: Uber health

After an eight-month trial with 100 health care providers that tested the ridesourcing service, Uber launched its new business line, Uber Health, in March 2018. Branded as a “HIPAA-compliant technology solution,” Uber Health provides a ridesourcing platform available specifically to health care providers, allowing clinics and hospitals to book rides for patients from a centralized dashboard. A health care associate inputs the name of the patient, a pick-up and drop-off location, and a phone number. The client then receives a text message or call with trip information at the time of booking and again when a driver is on the way. Rides can also be booked by clients with just a landline; they can be scheduled minutes before an appointment, or days in advance.

Uber Health stores all trip information in client-side, HIPAA-compliant servers, so organizations are able to view and export records for billing and reporting. Access to the Uber Health dashboard and reporting tools are free; Uber Health bills health care organizations directly for the cost of individual rides based on the same rate as rides on the standard consumer app. Uber has also created an open application programming interface so developers can build the service into their existing patient management software or health information technology systems.

### Type II: insurer partners with TNC

The second type of innovation we identified is when an insurer partners with a ridesourcing company. This is when a health plan or insurance company formally partners with an existing ridesourcing service(s) to expand transportation services available to beneficiaries or offer these transportation benefits for the first time. While examples of this type were limited, it is likely that this type of collaboration will become more common as insurers increasingly offer more supplemental, non-medical benefits as a result of a larger shift of the health care industry to value-based care. This is especially likely given the new Medicare Advantage guidelines for 2019 which make it easier for payers to receive compensation for providing a broader array of the supplemental benefits. Notably, this type of innovation reflects insurers’ acknowledgement of transportation to care as a social determinant of health.

### Examples of type II: Blue Cross and Blue Shield & Lyft; Cigna-HealthSpring & Lyft

In May 2017 Blue Cross and Blue Shield forged a public-private partnership with Lyft to address transportation challenges of some beneficiaries. Under the partnership, commercial plan members living in ‘transportation deserts,’ or areas with limited access to reliable transportation, can get a Lyft ride to medical appointments and the hospital at zero cost to them [22]. This offering was extended in 2018 to include rides to and from pharmacies, and further expanded in 2019 to members of certain Blue Cross and Blue Shield MA plans [23].

Also in May 2017, MA provider Cigna-HealthSpring partnered with Lyft to provide beneficiaries rides to physician offices, pharmacies, and health facilities. The service is for MA members in non-emergency situations and is only available to Cigna-HealthSpring customers with plans that have supplemental non-emergent medical transportation benefits through a program called Access2Care. By December 2017 the partnership had provided rides to 14,500 beneficiaries [24].

### Type III: Paratransit provider partners with TNC

The third type of innovation we identified is when a paratransit provider partners with a ridesourcing company. Due to the demand-responsive nature of paratransit provisions (e.g. services do not operate over a fixed schedule like a standard public bus; rather, vehicles are dispatched on request and operate door-to-door), paratransit services have been said to be a sort of progenitor of mobile app-based TNCs [25]. While not all paratransit trips are for health-related purposes, paratransit is especially important for people with disabilities who may have no other mode of reaching health care facilities or medical appointments. Sources that we located reference the increased flexibility and reliability of ridesourcing services compared to traditional paratransit. In most cases we found, transit agencies are subsidizing these trips while in a pilot phase, so long-term viability of these partnerships is unclear.

### Examples of type III: Massachusetts Bay Transportation Authority

A prominent example of this type of collaboration is led by the Massachusetts Bay Transportation Authority’s (MBTA) Paratransit service called the “RIDE.” Launched in September 2016 and extended three times (most recently through September 2020), the MBTA has been piloting a partnership program with Lyft, Uber, and now Curb, to offer on-demand service to eligible RIDE customers. Customers can enroll in the pilot program and sign up with one of the three TNCs (not multiple). Once enrolled, customers can request rides through Uber and Lyft smartphone apps or by using a call-in service if booking with Lyft or Curb. Riders receive a limited number of subsidized rides each month based on historical RIDE use.

According to MBTA, the maximum subsidy for each trip is currently $40. Uber and Lyft cover all of the RIDE’s regular service area while the newly added Curb service covers a smaller geographic subset of the area. Importantly, Uber, Lyft, and Curb drivers do not provide assistance (e.g. door to door service or help with vehicle boarding) in the same way that they would with the traditional RIDE service, so riders with these needs are encouraged to ride the traditional ADA Complementary Paratransit service [26].

Other regions have incorporated or piloted the use of TNC services for paratransit trips, including Broward Co. Paratransit in Broward County, Florida; Dallas Area Rapid Transit in Dallas, Texas; and RabbitTransit, the Central Pennsylvania Transportation Authority (formerly York Adams Transportation Authority).

## Discussion

In this environmental scan, we encountered various avenues through which innovation in shared mobility is driving the evolution of health care transportation. The on-demand nature of rides and integration of ride requests and electronic medical records (EMRs) appear to be the strongest drivers of this progress. Ridesourcing options are appearing in EMR workflows of clinicians and are becoming a part of the mode choice set for patients through formal partnerships with care providers, insurance companies, and transit agencies. Given the novelty of this type of collaboration, existing research on the topic is sparse. The environmental scan approach allows us to gather knowledge and identify shifts related to rapidly-evolving technological contexts as documented through news-based sources. Inherent to the nature of any keyword-based search strategy, our review is limited by the search terms we imposed and window of time we specified.

Press releases about new partnerships and mission statements on company websites speak of overarching goals driving this collaboration. Motivations are strong on the care provider side: increasing options for reliable patient transportation means reduction of no-shows and late arrivals, increased treatment adherence, and greater bed turnover as patients are discharged more swiftly. Cost saving potential for insurers is noteworthy; improving adherence to preventive care and maintenance of chronic conditions can reduce unnecessary emergency department visits. For patients, incentives focus largely on convenience. Ridesourcing options allow same-day, reliable access to urgent care and clinic appointments. In some cases, customer out of pocket expenses can be reduced (given the high cost of parking at some care facilities). For patients with physical limitations, ridesourcing services may offer greater freedom in scheduling medical trips; however, the accessibility of TNC vehicles for people with physical and intellectual disabilities remains a significant challenge to be addressed.

With the surge of innovation occurring in this space, there has been limited evaluation of effectiveness of ridesourcing interventions for medical trips and the evaluations that have occurred show mixed results. A 2017 study by researchers at the University of Kansas found that ambulance utilization decreased by an average of 7% from 2013 to 2015 in cities where UberX had been in operation [27]. A 2018 experiment by Penn Medicine researchers found that offering a free Lyft ride to medical appointments for Medicaid patients did not reduce the rate of missed primary care appointments in Philadelphia [28]. 

The efficacy of interventions designed to address transportation barriers, as well as interventions to address multiple social determinants of health, needs to be better understood. It is critical to consider whether new health care transportation options are equitable. Shared mobility users tend to be younger, have higher levels of educational attainment, and are less diverse than the general public and shared mobility modes often require access to a smartphone and banking services [29]. While several cases encountered in this scan are implementing avenues of utilization outside of traditional smartphone apps (e.g. dial-in options from a landline), further considerations should be explored to understand the reach of these services for various patient populations. Given the uncertainty around the future of TNC-based partnerships, research is needed to define best practices for collaborative management of these programs and to earnestly explore policies surrounding cross-sectoral data sharing feasibility.

## Conclusion

The fast pace of growth and innovation in the health care transportation sector reflects the longstanding need for progress in this area. With an aging baby boomer population, it is likely that the population of people reliant on external transportation provision to health care facilities will grow. The realization that transportation barriers to health care access are often preventable has dovetailed with the proliferation and familiarization of shared mobility technology in the US. Shared mobility can provide a viable option for populations with specific needs or barriers (e.g., older adults) and will likely continue its transformative impact on transportation access broadly [30].

Important shifts in health care delivery have contributed to creating an environment ripe for change. Movement towards value-based arrangements in the health care market, redistribution of financial risk of care, and new protection from sanctions for providing certain health-related services have made it permissible, and even judicious, for health care providers to offer or facilitate transportation for established patients.

Health care transportation, like the rest of the health care industry, is moving increasingly into the digital age. As patient medical records are progressively relocating online in the form of EMRs, so too are patient transportation arrangements shifting to web-based platforms. The findings from this scan are evidence that the benefits of connecting patients with on-demand rides to health care facilities through ridesourcing technology is deemed worth the potential risk of data breach or privacy concerns commonly associated with smart technologies. Continued innovation in this space must balance the goal of increased accessibility while prioritizing the protection of patient information.

Innovative health care mobility services aim to slow the chain reaction of missed appointments that trigger increased emergency room visits, extended hospital readmissions, higher overall costs, and poorer health outcomes. While new partnerships and companies continue to emerge in health care mobility services, it is critical for both health care providers and transportation providers to evaluate these offerings to ensure that they are accessible to the most vulnerable patient populations.

## Data Availability

The dataset used and/or analyzed during the current study are available from the corresponding author on reasonable request.

## Abbreviations

(ACOs): Accountable care organizations
(ADA): Americans with Disabilities Act
(EMRs): Electronic medical records
(MCOs): Managed care organizations
(MA): Medicare Advantage
(NEMT): Non-emergency medical transportation
(TNC): Transportation network company
(VA) (VA): U.S. Department of Veterans Affairs.

## Footnotes

1 Capitation is a payment arrangement for health care service providers; Medicaid pays the broker or MCO a set amount for each enrolled beneficiary assigned to them, per period of time, whether or not that person seeks care

2 and only when other means of transportation, such as a taxi or wheelchair van, would jeopardize the health of the beneficiary
